# The Impact of Diabetes Mellitus in Patients with Chronic Obstructive Pulmonary Disease (COPD) Hospitalization

**DOI:** 10.3390/jcm10020235

**Published:** 2021-01-11

**Authors:** Kulothungan Gunasekaran, Swetha Murthi, Kalaimani Elango, Mandeep Singh Rahi, Bright Thilagar, Sathishkumar Ramalingam, Dinesh Voruganti, Vijaya Kumar Paramasivam, Krishna Prasad Kolandaivel, Ashish Arora, Arul Chandran

**Affiliations:** 1 Division of Pulmonary Diseases and Critical Care, Yale-New Haven Health Bridgeport Hospital, 267 Grant Street, Bridgeport, CT 06610, USA; sunny.mandeep@gmail.com; 2 Division of Endocrinology and Metabolism, Lenox Hill Hospital, 110 e 59th Street, New York, NY 10022, USA; drswethamurthi@gmail.com; 3 Department of Cardiology, University of Nevada Las Vegas, 1701 W Charleston Blvd, Las Vegas, NV 89102, USA; kalaimani.elango@gmail.com; 4 Division of Hospital Medicine, Henry Ford Hospital, 2799 W Grand Blvd, Detroit, MI 48202, USA; bright.pearson@gmail.com; 5 Division of Hospital Medicine, Lovelace Medical Center, 601 Dr. Martin Luther King Jr. Ave NE, Albuquerque, NM 87102, USA; sathishmed@gmail.com; 6 Division of Cardiology, University of Arkansan Medical Sciences, 4301 W Markham Street, Little Rock, AR 72205, USA; dineshvoru@gmail.com; 7 Division of Nephrology, Bay State Medical Center, S 759 Chestnut Street, Springfield, MA 01199, USA; ronvijay@yahoo.com; 8 Division of Oncology, St. Joseph Medical Center, 523 N 3rd Street, Brainerd, MN 56401, USA; prasadarsenal87@gmail.com; 9 Division of Pulmonary Diseases and Critical Care, Saint Mary’s Hospital, 56 Franklin Street, Waterbury, CT 06610, USA; docashely@yahoo.co.uk; 10 Division of Pulmonary Diseases and Critical Care, Hurley Medical Center, G-3252 Beecher Road, Flint, MI 48532, USA; arulchandranmd@gmail.com

**Keywords:** COPD, chronic obstructive pulmonary disease, diabetes mellitus, length of stay, pneumonia, respiratory failure, stoke, kidney injury

## Abstract

(1) Background: Chronic obstructive pulmonary disease (COPD) is the leading cause of morbidity and mortality worldwide. Diabetes mellitus (DM) has been shown to have adverse inflammatory effects on lung anatomy and physiology. We investigated the impact of DM on COPD patient outcomes during inpatient hospitalization. (2) Methods: We conducted a retrospective analysis using the Nationwide Inpatient Sample (NIS) over the years 2002–2014. Three groups, COPD without diabetes, COPD with diabetes but no complication, and COPD with DM and complication, were analyzed. (3) Results: A total of 7,498,577 were COPD hospitalization; of those, 1,799,637 had DM without complications, and 483,467 had DM with complications. After adjusting for clinical, demographic, and comorbidities, the odds of increased LOS in the COPD/DM with complication were 1.37 (confidence interval (CI): 1.326–1.368), and those of DM without complication were 1.061 (1.052–1.070) when compared with COPD alone. The odds of pneumonia, respiratory failure, stroke, and acute kidney injury were also higher in COPD hospitalizations with DM. Both DM with complication (odds ratio (OR): 0.751 (CI 0.727–0.777)) and DM without complication (OR: 0.635 (CI: 0.596–0.675)) have lesser odds of mortality during hospitalization than with COPD alone. (4) Conclusions: There is a considerable inpatient burden among COPD patients with DM in the United States.

## 1. Introduction

Chronic obstructive pulmonary disease (COPD) is a chronic inflammatory lung disease that causes progressive airflow obstruction, which is not fully reversible. The airflow limitation is associated with an abnormal inflammatory response of the lung to noxious particles or gases. According to the Global Initiative for Chronic Obstructive Lung Disease (GOLD) 2020 report, COPD is the leading form of lung disease causing morbidity and mortality worldwide. It is associated with a substantially increasing economic and social burden [1,2]. According to a systematic review and meta-analysis published by Adiloye et al. in 2015, there were about 384 million COPD cases in 2010, with an estimated global prevalence of around 11.7% [3]. Globally, there were about 3 million deaths due to COPD annually [4]. With the increase in the prevalence of smoking and aging in the patient population, the prevalence of COPD is projected to rise over the next 40 years. There may be more than 5.4 million annual deaths from COPD and related conditions in 2060 [5].

Studies have shown that morbidities in COPD increase with age [6] and are also affected by other comorbid conditions like diabetes and cardiovascular diseases [7]. These chronic conditions impair the patient’s health status and affect COPD management, adding to the economic and social burden.

Diabetes is one of the leading causes of morbidity and mortality in the world. It was the seventh leading cause of death in the United States in 2017. According to the Centers for Disease Control and Prevention (CDC) 2020 report, about 34.2 million people have diabetes, which is about 10.5% of the U.S. population, and 88 million people aged 18 years and older have prediabetes (about 34.5% of U.S. adult population) [8]. About 1.5 million Americans are diagnosed with diabetes every year.

Diabetes is a metabolic disorder characterized by hyperglycemia, resulting from insulin production defects, action, or both. Chronic hyperglycemia is associated with dysfunction and long-term damage to various organs such as the kidneys, eyes, heart, and blood vessels. Studies have shown that there is an association of inflammation with diabetes where there is an increase in the circulating levels of inflammatory markers, including C reactive protein (CRP), interleukin-6 (IL-6), and fibrinogen, which lead to insulin resistance [9]. This chronic systemic inflammation forms the most common denominator for COPD and DM. Several studies have shown that diabetes has a significant implication on the respiratory condition, including COPD, obstructive sleep apnea, and interstitial lung disease [10,11,12]. Diabetes mellitus (DM) in COPD patients was found to affect their lung function and, in turn, their prognosis [13,14], but their impact on COPD hospitalizations in terms of pneumonia, acute respiratory failure, stroke, acute kidney injury, and inhospital mortality has not been studied. COPD patients with comorbid DM are shown to have an increase in their length of stay [14]. Identifying the factors that affect the length of stay and charges incurred during the hospital stay can help provide better insight to the physicians regarding managing these chronic conditions. Given the association between COPD and DM, we investigated DM’s impact on COPD outcomes during inpatient hospitalization.

## 2. Materials and Methods

### 2.1. Data Source

The data that support the findings of this study are available from the corresponding author on reasonable request. The study examined the impact of diabetes mellitus (DM) on COPD hospitalizations using discharge data from the National Inpatient Sample (NIS), Healthcare Cost and Utilization Project (HCUP), and Agency for Healthcare Research and Quality [15]. The NIS is the largest publicly available all-payer inpatient care database in the United States (US), containing data on more than seven million hospital stays per year. NIS is a self-weighted, stratified, systematic, random sample of 20% discharges from all non-federal U.S. community hospitals (before 2012, it was a 20% sample of hospitals from which all discharges were retained). To account for this redesign, the trend weight (TRENDWT) provided by HCUP is used in place of the original discharge weight (DISCWT) to create national estimates for years before 2012 [16]. The NIS sample is stratified on hospital characteristics. This clustering form tends to induce dependence among discharges within hospitals; hence, variance analysis of subsets in line with NIS methods [17] was performed. Because this study included de-identified data, per the data use agreement with the Agency for Healthcare Research and Quality, ethical clearance and patient consent were not sought.

### 2.2. Study Population and Variables

We used *International Classification of Diseases, Ninth Revision, Clinical Modification* (*ICD-9-CM*) codes to identify all hospitalized adults (aged ≥ 18 years) who had a primary diagnosis of COPD (*ICD-9-CM* diagnosis codes 491.2, 491.20, 491.21, 491.22, 492, 492.0, 492.8, 496) between January 2002 and December 2014. Those with missing data for gender, mortality, and length of stay were excluded. Those coded for “DISPUNIFORM = Transfer to Short-term Hospital” and “Admission source, uniform coding—ASOURCE = transfer from another hospital” for the years 2002–2007 and “Indicator of a transfer into the hospital—TRAN_IN = Transferred in from a different acute care hospital” for the years 2008–2014 were also excluded to avoid duplication of discharges, which yielded a final sample size of 7,498,577. Dummy cases with hospital identifiers were added to ensure all the hospitals in the United States, irrespective of COPD diagnosis, were added to the analysis to account for the NIS database’s intricate sampling design considering the clustering effect.

Among COPD admissions, those with DM were extracted using the clinical classification of diseases software (CCS) code [18] from the secondary diagnoses numbers *DXCCS2-DXCCS30*. CCS code 49 is used for DM without complication, CCS code 50 is used for DM with complications, and those without any of the above codes were named “COPD with no DM”. DM with complications includes patients with DM and complications like ketoacidosis, hyperosmolarity, coma, renal, ophthalmic, neurological, or peripheral vascular disease manifestation. NIS provides 29 comorbidities (also known as Elixhauser comorbidity measures) based on *ICD-9 CM* diagnoses and the diagnosis-related group in effect on the date of discharge. These comorbidities are not directly related to the principal diagnosis or the main reason for admission and are likely to have originated before the hospital stay [19]. Additional comorbidities and clinical outcomes were extracted using *ICD-9-CM* diagnosis and procedure codes described in the Supplementary Table S1.

### 2.3. Outcomes

Our study aimed at finding the differences in outcomes such as hospital length of stay, the incidence of pneumonia, respiratory failure, acute kidney injury, and stroke among three subgroups of COPD inlcuding COPD with no DM, COPD with DM without complication, and COPD with DM with complication.

### 2.4. Statistical Analyses

We adhered to the methodological standards described by Khera et al. [20]. All data analyses were performed using IBM SPSS Statistics for Windows, version 24.0 (IBM, Armonk, New York, NY, USA). The study analysis was done using a complex sample analysis method accounting for the sample design’s clustering effect. Weight is applied to obtain national estimates. Continuous values are reported as mean ± standard error of the mean and compared using analysis of variance (ANOVA). Categorical variables were reported as a number and/or percentage and compared using the Chi-square test. Multivariable logistic regression analyses were done while accounting for the sampling technique and adjusting for various demographic variables, including age, clinical variables, and hospital characteristics.

## 3. Results

Out of 7,498,577 (sample size N) patients with COPD over 18 years, 521,5474 (69.55%) did not have diabetes. Of those remaining patients who had diabetes (*n* = 2,283,103), about 21.18% of patients (*n* = 483,467) had complications due to diabetes. There was more female representation in the sample population; about 55.8% of those with COPD without DM were females. The mean age of all COPD patients was 68.92 years (interquartile range (IQR) 68.86, 68.98 years). Of those patients without DM, the mean age was about 69.17 ± 0.035. Compared with those without DM complications, patients with DM complications were younger (68.57 ± 0.036 vs. 67.55 ± 0.051). Among all COPD admissions, about 66.8% were Caucasians, followed by African-Americans (8.1%). The ethnic distribution was similar among those patients with no DM (67.7% and 7.2% respectively), DM with no complications (65.2% and 9.8% respectively), and DM with complications (63.1% and 11.5% respectively). About 30.3% of COPD patients without DM were current smokers compared with 26% and 24% in diabetic patients without complications and with complications, respectively (*p* < 0.001) (Table 1).

Among all COPD patients who had approximately nine comorbid medical conditions, patients with DM and no complications had about ten chronic medical conditions. Those with DM complications had about 12 comorbidities. The typical chronic medical conditions included hypertension, congestive heart failure, obesity, fluid and electrolyte abnormalities/renal failure, deficiency anemias, depression, and hypothyroidism.

About 72.6% of the diabetic complications had hypertension compared with 53.6% in patients without diabetes. Congestive heart failure was also more common among diabetic patients with complications than those without diabetes (40% and 21.3% respectively, *p*-value < 0.0001). Similarly, obesity was also more common in diabetic patients with complications than those without diabetic complications and those without diabetes (28.2% vs. 19% vs. 6.6%, respectively).

Of diabetic patients with complications, about 23% had renal failure, and 27.9% had fluid and electrolyte abnormalities. In comparison, in the non-diabetic patient group, 6.5% had renal failure and 21.7% had fluid and electrolyte abnormalities.

About 15.4% of diabetic patients with complications were diagnosed with depression, compared with 14.1% in patients without diabetes. It was a statistically significant (<0.0001) difference between the two groups, even though the absolute difference was small.

### 3.1. Prevalence

**Figure 1:**
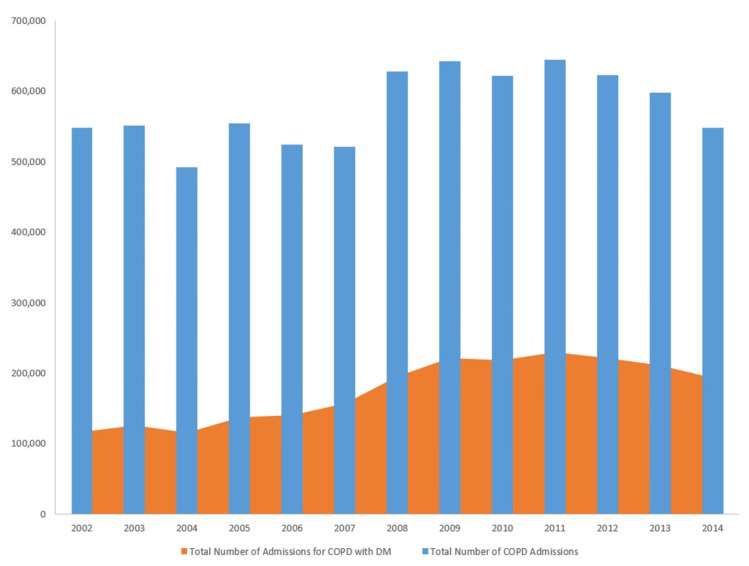
Trends of hospitalization for chronic obstructive pulmonary disease (COPD) and diabetes mellitus (DM) in COPD 2005–2014.

Our study results showed that diabetes mellitus was increasingly prevalent in patients with COPD hospitalizations for over ten years (Table 2) (Figure 1).

### 3.2. Length of Stay

When the length of stay (with ≥4 days of hospital stay) was compared among the group, about 51% of patients without diabetes had a longer length of stay. As expected, about 60.8% of diabetic patients with complications had a longer length of stay compared with 53.4% of diabetic patients without complications (*p* < 0.001). After adjusting for clinical, demographic characteristics, and comorbidities, logistic regression analysis showed that patients with DM, but no complications had an odds of 1.061 (95% confidence interval (CI) 1.052, 1.070) risk of having an extended hospital stay ≥4 days. Diabetic patients with complications had an increased odds of 1.347 (95% CI 1.326, 1.368) of having an extended hospital stay ≥4 days compared with patients without DM. This was evident in the total charges incurred during the hospital stay among the patient population. Those patients without diabetes had a total cost of about $21,321.90 (95% CI 21,043.69, 21,600.11). This was much lower than the total charges incurred for the diabetic patients without complications and those with complications ($23,080.01 ± SE 154.647 vs. $27,328.29 ± SE 234.908, *p*-value < 0.0001).

### 3.3. Outcomes

Logistic regression was performed to assess the odds of developing pneumonia, respiratory failure, acute kidney injury, stroke, and mortality after adjusting for clinical, demographic characteristics, and comorbidities (Table 3). The odds of pneumonia were statistically significant between diabetic patients with complications (odds ratio (OR) 1.04, 95% CI 1.02, 1.05) and those with diabetes without complications (OR 1.07, 95% CI 1.06, 1.08) when compared with COPD without diabetes. The patients’ odds of developing respiratory failure were slightly higher in diabetic patients without complications and with complications compared with those without diabetes (1.05 and 1.05, respectively). When compared with patients with COPD and no diabetes, patients with COPD and DM without complications had an odds ratio of 1.086 for acute kidney injury (95% CI 1.071–1.010) (*p* < 0.0001) COPD and DM with complications had an odds ratio of 1.594 (95% CI 1.567–1.622) (*p* < 0.0001). The odds of developing stroke during inpatient admission was not statistically different between COPD without diabetes and COPD and diabetes without complications. There is a statistically significant increased odds of getting stroke among COPD and diabetes with complications compared with COPD alone (OR 1.17, 95% CI 1.13, 1.22). Surprisingly, both diabetic patients without complications (OR 0.751 95% CI 0.727, 0.777) and those with complications (OR 0.635, 95% CI 0.596, and 0.675) had lower odds of mortality than those without diabetes.

Among all the variables, age is a significant factor that can impact mortality. We conducted a multivariate analysis where age was found to have higher odds of mortality independent of DM and other comorbidities with an adjusted odds ratio of 1.033, i.e., for every one-year increase in age, the odds of dying increase by 3.3% (Table 4).

## 4. Discussion

COPD and DM are chronic medical conditions that individually affect the quality of life of patients. If present together, they can further complicate patient management. The impact of these chronic inflammatory conditions has always been an area of interest and public health concern. Observational studies play an essential role in formulating a hypothesis and provide the basis for understanding long-term comorbidities and their association with other medical conditions. Our study provides an insight into understanding the effect of pre-existing DM morbidity, healthcare cost, and mortality of COPD patients. In our study, about 30.45% of COPD patients had pre-existing DM, of which the study population had greater female representation compared with males. Recent studies have shown that women were more likely than men to have COPD diagnostic delays, inadequate quality of care, a higher frequency of severe exacerbations, a higher number of hospitalizations, and prolonged length of stay for hospitalization [21,22,23]. Our population also showed women preponderance. Our sample population’s mean age was about 68.92 years, almost similar to the prior cohort studies [24].

The longer length of stay, defined as a hospital stay ≥4 days, was higher in diabetic patients with complications than those without diabetes. This was also inferred in the total hospital charges, which were inflated for those patients with diabetes complications. This was seen even in an emergency department (ED) observation unit, as mentioned in a retrospective cohort study conducted in Thailand, where the patients with diabetes had increased odds of prolonged stay at the emergency department >48 h or COPD-related ED revisit within 72 h or readmission within one month [25].

Our study showed that COPD with diabetes has an increased risk of pneumonia and respiratory failure. Our results are similar to the study performed by Ehrlich et al. Their observation between diabetes and COPD showed an increased occurrence of pneumonia in patients with diabetes and decreased pulmonary function related to hyperglycemia [26]. As hyperglycemia is associated with significant lung function decline, we speculate that it could be the reason our COPD hospitalizations with diabetes had higher odds of respiratory failure [27,28,29].

The limited amount of epidemiological literature on this topic has identified the association of acute kidney disease with COPD. A retrospective cohort study using electronic medical records in Taiwan showed that COPD is associated with a higher risk of acute kidney injury development [30]. Another study showed the possible mechanism of acute kidney injury might probably be due to diuretics and contrast media [31]. Further, pathophysiological changes of acute kidney injury under high-glucose status might be due to oxidative stress and increased reactive oxygen species, which cause stronger vessel constriction and insufficient oxygen supply in the kidney via vasoactive substances [32]. Similar to other studies, our study showed that diabetes with complications had much higher odds of acute kidney injury than diabetes without complications on COPD hospitalizations.

In our analysis, we found increased odds of stroke among COPD hospitalizations and diabetes with complications. In general, the association between COPD and stroke is dependent on the other traditional stroke risk factors apart from smoking. COPD and diabetes can cause systemic inflammation, and oxidative stress may play an essential role by promoting cerebral vascular dysfunction and platelet hyperactivity [33,34]. Previous studies have shown that reduced FEV1 is associated with an increased incidence of both ischemic and hemorrhagic stroke, and this association is independent of smoking status [35,36]. Recent meta-analysis also showed that increased HbA1C is associated with increased prevalence and poor outcomes of stroke among the diabetic patient population [37]. Our study has shown that the combination of COPD and diabetes would increase the odds of stroke, and the odds ratio would be greater with concurrent complications.

Our study found that diabetic patients with and without complications had lower odds of mortality than those without diabetes. This contrasts with a cohort study published by Te-Wei Ho et al. [11]. This study showed that pre-existing DM in COPD patients had a higher hazard ratio (HR) for mortality than those without DM. This could be because of the small sample size and possible mortality, because the majority of the patient population (about 46%) were more than 70 years of age. There are potential explanations for our findings. In our data set, COPD hospitalizations with DM were relatively younger and might have a higher body mass index than the COPD patients with no diabetes. Several studies have shown that underweight patients had a higher mortality than COPD patients with normal BMI, whereas overweight and obese individuals were associated with lower mortality [38,39,40,41,42]. In our study, we found that obesity was more common in diabetic patients with complications, and they had lower odds of mortality. It is possible the obesity might have a protective effect against mortality in these patients.

Age alone is a risk factor for mortality in COPD patients, which is evident from our analysis. Kim et al. showed that, after grouping the population by age quartiles, the rate of FEV1 decline was faster among older patients than younger ones [43]. Other studies have also shown that COPD patients had higher odds of inpatient mortality and morbidity, particularly if they were more than 80 years of age [44,45]. So, we included age in the logistic regression as a confounder. After adjusting for all the confounders, including age, mortality was still lower in both groups of diabetes compared with COPD with no diabetes.

### 4.1. Strengths

The large sample size is the study’s major strength, representing almost 95% of U.S. hospitalizations. Our study is the first direct evidence regarding the association between diabetes and health outcomes among COPD hospitalizations in the United States.

### 4.2. Study Limitations

The use of administrative databases has certain limitations. First, because this is a cross-sectional observational study, the possibility of selection bias and residual measured and unmeasured confounding cannot be excluded entirely. Second, as NIS is an administrative database, the data’s consistency and accuracy depend mainly on the coder’s experience and the clinician’s documentation. Hence, there may have been a possibility of some variation in the data because of the unrecognized miscoding of diagnostic codes. However, these potential limitations may be compensated for by the large size of the database and the ability to obtain nationwide estimates using the discharge weights provided. Another significant limitation is that NIS is a database of discharges; hence, individual patient analysis, such as the medications, cause of exacerbation (viral vs. bacterial), cause of death, or body mass index, cannot be measured. Besides, we lack information such as pulmonary function tests (PFTs) and the severity of COPD.

## 5. Conclusions

In conclusion, diabetes is a highly prevalent comorbidity among COPD hospitalizations and is associated with poorer outcomes than COPD hospitalizations without diabetes. If diabetes is associated with complications, there is a strong association with an increased length of stay, increased risk of stroke, and acute kidney injury. Our study highlights the importance of better control of diabetes, particularly in chronic conditions like COPD, to minimize life-threatening complications.

## Figures and Tables

**Table 1:** Demographic characteristics of chronic obstructive pulmonary disease (COPD) hospitalizations with and without diabetes mellitus (DM).

Clinical Characteristics	All COPD (*n* = 7,498,577)	COPD with No DM (*n* = 5,215,474)	COPD with DM without Complication (*n* = 1,799,636)	COPD with DM with Complication (*n* = 483,467)	*p*-Value *
Age (years)	68.92 ± 0.03	69.17 ± 0.03	68.57 ± 0.03	67.55 ± 0.05	<0.0001
Sex					
Male ^†^	44.1%	69.8%	23.9%	6.3%	<0.0001
Female ^†^	55.9%	69.4%	24.1%	6.5%	
Race (uniform)					
White	66.8%	67.7%	65.2%	63.1%	<0.0001
Black	8.1%	7.2%	9.8%	11.5%	
Hispanic	3.9%	3.3%	4.7%	6.4%	
Asian or Pacific Islander	0.8%	0.8%	0.9%	0.9%	
Native American	0.4%	0.4%	0.5%	0.5%	
Others	1.4%	1.3%	1.5%	1.7%	
Missing	18.6%	19.3%	17.4%	16.0%	
Current smoking	28.8%	30.3%	26.0%	24.0%	<0.0001
Past smoking	20.0%	19.8%	20.6%	19.5%	<0.0001
Admission day is a weekend	23.9%	23.8%	24.1%	23.9%	<0.0001
Disposition of patient (uniform)					
Home or self-care	67.3%	68.0%	66.5%	63.0%	<0.0001
Skilled nursing facility (SNF)	14.3%	14.2%	14.4%	15.1%	
Home health care (HHC)	15.3%	14.5%	16.6%	19.6%	
Against medical advice (AMA)	1.3%	1.3%	1.2%	1.2%	
Died in hospital	1.7%	1.9%	1.3%	1.2%	
Unknown	0%	0.1%	0%	0%	
Elective versus non-elective admission					
Non elective	91.9%	91.7%	92.2%	92.8%	<0.0001
Elective	8.1%	8.3%	7.8%	7.2%	
Number of comorbidities	9.23 ± 0.02	8.46 ± 0.02	10.63 ± 0.02	12.40 ± 0.04	<0.0001
Comorbid conditions					
Acquired immune deficiency syndrome	0.2%	0.3%	0.1%	0.1%	<0.0001
Alcohol abuse	3.8%	4.4%	2.5%	2.1%	<0.0001
Deficiency anemias	13.7%	12.5%	15.6%	19.8%	<0.0001
Rheumatoid arthritis/collagen vascular diseases	2.7%	2.7%	2.6%	2.4%	<0.0001
Chronic blood loss anemia	0.5%	0.5%	0.5%	0.6%	<0.0001
Congestive heart failure	25.0%	21.3%	31.8%	40.0%	<0.0001
Coagulopathy	2.2%	2.1%	2.3%	2.9%	<0.0001
Depression	14.3%	14.1%	14.9%	15.4%	<0.0001
Drug abuse	2.3%	2.5%	1.9%	2.1%	<0.0001
Hypertension	58.5%	53.6%	69.0%	72.6%	<0.0001
Hypothyroidism	11.4%	10.9%	12.5%	13.5%	<0.0001
Liver disease	1.7%	1.6%	1.8%	2.4%	<0.0001
Lymphoma	0.5%	0.6%	0.4%	0.4%	<0.0001
Fluid and electrolyte disorders	22.1%	21.7%	21.8%	27.9%	<0.0001
Metastatic cancer	1.2%	1.4%	0.9%	0.8%	<0.0001
Other neurological disorders	6.9%	7.0%	6.7%	7.0%	<0.0001
Obesity	11.0%	6.6%	19.0%	28.2%	<0.0001
Paralysis	1.1%	1.0%	1.1%	1.3%	<0.0001
Peripheral vascular disorders	7.3%	6.8%	7.6%	11.1%	<0.0001
Psychoses	5.1%	4.8%	5.5%	5.8%	<0.0001
Pulmonary circulation disorders	0.4%	0.4%	0.5%	0.6%	<0.0001
Renal failure	8.7%	6.5%	11.2%	23.0%	<0.0001
Hemodialysis	0.9%	0.7%	0.9%	2.8%	<0.0001
Solid tumor without metastasis	3.3%	3.5%	2.7%	2.2%	<0.0001
Peptic ulcer disease excluding bleeding	0.2%	0.2%	0.1%	0.1%	<0.0001
Valvular disease	5.6%	5.5%	5.6%	6.1%	<0.0001
Weight loss	3.2%	3.7%	2.0%	2.1%	<0.0001
Atrial fibrillation and flutter	14.4%	13.7%	16.0%	16.7%	<0.0001
Coronary artery disease	30.2%	27.0%	36.6%	41.1%	<0.0001
Smoking	42.5%	44.2%	39.1%	36.6%	<0.0001
Hyperlipidemia	27.4%	23.2%	36.3%	40.8%	<0.0001
Obstructive sleep apnea	8.5%	5.4%	14.3%	19.5%	<0.0001
Marijuana	0.3%	0.3%	0.2%	0.2%	<0.0001
Outcomes					
Died during hospitalization	1.7%	1.9%	1.3%	1.2%	<0.0001
Pneumonia	14.0%	13.8%	14.5%	14.4%	<0.0001
Respiratory failure	9.2%	8.7%	10.0%	11.4%	<0.0001
Stroke	1.0%	0.9%	1.0%	1.2%	<0.0001
Sepsis	1.0%	1.0%	0.9%	1.4%	<0.0001
ET intubation/mechanical ventilation	2.3%	2.3%	2.2%	2.5%	<0.0001
Acute kidney injury	4.5%	3.7%	5.3%	11.0%	<0.0001
Resource utilization					
Length of stay (LOS in days)	4.58 ± 0.009	4.52 ± 0.01	4.56 ± 0.01	5.22 ± 0.02	<0.0001
LOS ≥ 4 days	52.2%	51.0%	53.4%	60.8%	<0.0001
Total charges	22,131.10 ± 141.89	21,321 ± 141.93	23,080 ± 154.64	27,328 ± 234.90	<0.0001

Categorical variables are displayed as mean ± standard error of the mean. Continuous variables are expressed as %. * = all *p*-values denote the comparison of each variable across three subgroups of COPD. Continuous variables across three subgroups are compared by analysis of variance (ANOVA), and categorical variables are compared by Pearson Chi-square test. ^†^ = percentages are derived by taking either male or female as a whole group for the three subgroups.

**Table 2:** Trends of hospitalization for chronic obstructive pulmonary disease and diabetes mellitus in COPD 2005–2014.

Calendar Year	Total Number of COPD Admissions	Total Number of Admissions for COPD with DM	Inpatient Prevalence of DM (per 1,000,000 Admissions) among COPD Patients
2002	548,528	116,211	211,860
2003	551,403	126,200	228,872
2004	492,379	115,465	234,503
2005	554,147	137,344	247,849
2006	524,685	140,828	268,405
2007	521,399	156,782	300,695
2008	628,521	195,251	310,652
2009	642,172	221,822	345,424
2010	621,362	218,531	351,697
2011	644,382	229,464	356,100
2012	622,720	221,365	355,481
2013	598,379	210,595	351,942
2014	548,500	193,245	352,315
Total	7,498,577	2,283,104	304,472

**Table 3:** Multivariate logistic regression analysis showing the adjusted odds ratios predicting the specific outcomes for chronic obstructive pulmonary disease hospitalizations.

Outcomes	DM without Complication vs. No DM				DM with Complication vs. No DM			
	Odds Ratio	95% Confidence Interval			Odds Ratio	95% Confidence Interval		
		Lower	Upper	*p*-Value		Lower	Upper	*p*-Value
LOS ≥ 4 days	1.06	1.06	1.07	<0.0001	1.40	1.39	1.41	<0.0001
Pneumonia	1.07	1.06	1.08	<0.0001	1.04	1.02	1.05	<0.0001
Respiratory failure	1.05	1.04	1.07	<0.0001	1.05	1.04	1.07	<0.0001
Acute kidney injury	1.086	1.071	1.100	<0.0001	1.594	1.567	1.622	<0.0001
Stroke	1.018	0.994	1.042	0.143	1.179	1.136	1.225	<0.0001
Inhospital mortality	0.79	0.77	0.80	<0.0001	0.71	0.68	0.74	<0.0001

**Table 4:** Multivariate logistic regression analysis showing the adjusted odds ratios of age predicting the mortality for chronic obstructive pulmonary disease hospitalizations.

Variable	Odds Ratio for Mortality	95% Confidence Interval		*p*-Value
Age in years at admission	1.033	1.031	1.035	<0.0001

## Data Availability

The data presented in this study are available on request from the corresponding author.

## Supplementary Materials

The following are available online at https://www.mdpi.com/2077-0383/10/2/235/s1, Table S1: ICD codes.
