# Supplementary material for: The Impact of Diabetes Mellitus in Patients with Chronic Obstructive Pulmonary Disease (COPD) Hospitalization
Source: J Clin Med. 2021 Jan 11;10(2):235. doi: 10.3390/jcm10020235 (PMC7827207; doi:10.3390/jcm10020235)
Supplement: Supplementary file 1 [file jcm-10-00235-s001.pdf]

Diagnosis	Diagnoses - ICD 9 CM codes	Procedures -ICD 9 CM Codes	Diagnoses - Clinical Classification Software (CCS) codes
COPD	491.2,491.20,491.21,491.22,492,492.0,492.8,496		
Diabetes mellitus without complication			49
Diabetes mellitus with complications			50
Pneumonia	480,480.1,480.2,480.3,480.8,480.9,481,482,482,482.1, 482.2,482.3,482.30,482.31,482.32,482.39,482.4,482.4, 482.41,482.41,482.42,482.49,482.8,482.81,482.82,482.83,482.84,482.89,482.9,483,483,483.1,483.8,484,484.1, 484.3,484.5,484.6,484.7,484.8,485,486,487,487,487, 488.01,488.11,488.81		
Respiratory failure	518.81,518.82,799.1,V46.1,V46.11	93.9,96.04,96.05,96.7,96.71,96.72	
Current smoking	305.1,305.1,305.11,305.12,305.13,649,649.01,649.02, 649.03,649.04,989.84		
Past smoking	V15.82		
Marijuana	304.30,304.31,304.32,305.20,305.21,305.22		
Atrial fibrillation & flutter	427.31,427.32		
Known CAD	414.00,414.01,414.02,414.03,414.04,414.05,414.06, 414.07,414.0,412,413.0,413.9,414.2,414.3,414.4,414.8, 414.9,V45.82,V45.81		

Sepsis	003.1, 003.21, 020.2, 022.3, 036.0, 036.1, 036.2, 036.3, 036.42, 038.0, 038.1, 038.10, 038.11, 038.19, 038.2, 038.3, 038.4, 038.40, 038.41, 038.42, 038.43, 038.44, 038.49, 038.8, 038.9, 098.82, 098.84, 098.89, 112.5, 112.81, 112.83, 115.04, 115.14, 115.94, 117.9, 785.52, 790.7, 995.92		
Mechanical ventilation	96.04; 96.05 96.7; 96.70; 96.71; 96.72		
Hemodialysis		39.95	
Acute kidney injury	584,584.5,584.6,584.7,584.8,584.9		
Shock(septic+cardiogenic)	785.5,785.59,785.51,785.52		
Respiratory arrest	799.1		
Hyperlipidemia	272.0,272.1,272.2,272.3,272.4,272.5		
Stroke	431,433,433.0,433.00,433.01,433.1,433.10,433.11,433.2,433.20,433.21,433.3,433.30,433.31,433.8,433.80,433.81,433.9,433.90,433.91,434,434.0,434.00,434.01,434.1,434.10,434.11,434.9,434.90,434.91,436,437.1,997.02		
Obstructive sleep apnea	327.20,327.23,327.29,780.51,780.53,780.57		
